# Evaluation of machine learning techniques for real-time prediction of implanted lower limb mechanics

**DOI:** 10.3389/fbioe.2024.1461768

**Published:** 2025-01-15

**Authors:** Chase Maag, Clare K. Fitzpatrick, Paul J. Rullkoetter

**Affiliations:** ^1^ DePuy Synthes, Warsaw, IN, United States; ^2^ Department of Mechanical and Biomedical Engineering, Boise State University, Boise, ID, United States; ^3^ Center for Orthopaedic Biomechanics, University of Denver, Denver, CO, United States

**Keywords:** machine learning, total knee replacement, kinematics, kinetics, finite element, computational biomechanics

## Abstract

**Introduction:**

Accurate prediction of knee biomechanics during total knee replacement (TKR) surgery is crucial for optimal outcomes. This study investigates the application of machine learning (ML) techniques for real-time prediction of knee joint mechanics.

**Methods:**

A validated finite element (FE) model of the lower limb was used to generate a dataset of knee joint kinematics, kinetics, and contact mechanics. The models were trained on joint alignment data, ligament information, and external boundary conditions. Several predictive algorithms were explored, including linear regression (LRM), multilayer perceptron (MLP), bi-directional long short-term memory (biLSTM), convolutional neural network (CNN), and transformer-based approaches. The performance of these models was evaluated using average normalized root mean squared error (nRMSE).

**Results:**

The biLSTM model achieved the highest accuracy, with a significantly lower nRMSE compared to other models. Compared to traditional FE or rigid body dynamics models, these predictive models offered significantly faster prediction speeds, enabling near-instantaneous insights into the TKR system’s performance. The small size of the predictive models makes them suitable for deployment on edge devices potentially used in operating rooms.

**Discussion:**

These findings suggest that real-time biomechanical prediction using biLSTM models has the potential to provide valuable feedback for surgeons during TKR surgery. Applications of this work could be applied to provide pre-operative guidance on optimal target implant alignment or given the real-time prediction ability of these models, could also be used intra-operatively after integration of patient-specific intra-op kinematic and soft-tissue information.

## Introduction

Total knee replacement (TKR) is a surgical procedure to replace a knee joint damaged by disease or injury with prosthetic components. TKR is a common procedure, with over a million performed annually in the United States (Choi and Ra, 2016). Preclinical evaluations of TKR devices are indispensable to ensure their reliability and effectiveness prior to patient use. Various approaches such as *in vitro* experiments along with computational modeling can be employed for this purpose. Such preclinical studies facilitate the early detection of potential issues regarding implant design or positioning under physiological stresses (Maletsky and Hillberry, 2005; Willing et al., 2019; Behnam et al., 2024). They contribute to enhancing implant design and evaluation of the kinematics, contact mechanics, and potential longevity of the device under various conditions.

Computational models have been used for many years to contribute to preclinical design iterations of total knee replacement implants (Knight et al., 2007; Baldwin et al., 2009; Halloran et al., 2010; Harris et al., 2016; Andreassen et al., 2021; Loi et al., 2021). Studies have focused on a variety of topics, including implant design, surgical decisions, and subject-specific factors (Dhaher and Kahn, 2002; Mesfar and Shirazi-Adl, 2005; Kessler et al., 2008; Elias et al., 2010; Thompson et al., 2011; Willing and Kim, 2011; Galloway et al., 2012). Probabilistic studies have incorporated variation in external boundary conditions, surgical alignments, and ligamentous changes to better capture subject-specificity and population variability in these computational models (Kebbach et al., 2023; Rothammer et al., 2023). Fitzpatrick et al. (2012), Fitzpatrick et al. (2014b), Fitzpatrick et al. (2014a) quantified the relative contributions of surgical, design, and patient variability to the overall variability in joint mechanics.

Although very important for preclinical development, the typical analysis time required to run a single complex simulation (typically in the order of ∼1–12 h, depending on model complexity and available computing resources) has limited their real-time use in other applications, such as intra-operative, patient-specific decision making to determine ideal implant alignment. In this setting, it is essential to have instantaneous access to the impact of patient ligament balance and implant alignment on estimated joint mechanics. One recent study effectively developed a statistical shape-function model to instantaneously predict output knee mechanics from implant alignment and design parameters using linear regression analysis (Gibbons et al., 2019). However, this study focused on a simplified knee joint and did not consider patient-specific ligamentous laxity. Given the recent advancements in developing machine learning methods for time series applications and predictions (Fawaz et al., 2019; Zerveas et al., 2021), as well as rapid increases in computational capabilities; the development of real-time predictive time series biomechanics models in becoming increasingly viable and so is garnering interest from the biomechanics community.

Mansour et al. investigated the accuracy of several predictive techniques on the ability to predict joint moments at the ankle, knee and hip joint during sit-to-stand (Mansour et al., 2023). These machine learning and linear regression models allow for real-time prediction of knee joint biomechanics that can help inform pre-operative and intra-operative decision making for an idealized individual approach (Dossett et al., 2024).

The objective of this study was to investigate time series prediction techniques for implanted knee joint biomechanics with varied surgical alignments, loading, and collateral ligament conditions. Inputs to the predictive tools included 7 implant alignment parameters and 7 external boundary and loading conditions, while 39 TKR-implanted lower limb parameters were predicted (kinematics, kinetics, contact mechanics, muscle forces, and ligament tensions). The methods evaluated ranged from simple linear regression modeling (LRM) to more complex machine learning (ML) techniques: multilayer perceptron (MLP), bi-directional long-short term memory (biLSTM), convolutional neural network (CNN), and transformer based approaches. Identification of a reliable predictive method may have applications in efficient pre-clinical testing, design optimization, and intra-operative decision-making.

## Methods

### Summary

**FIGURE 1:**
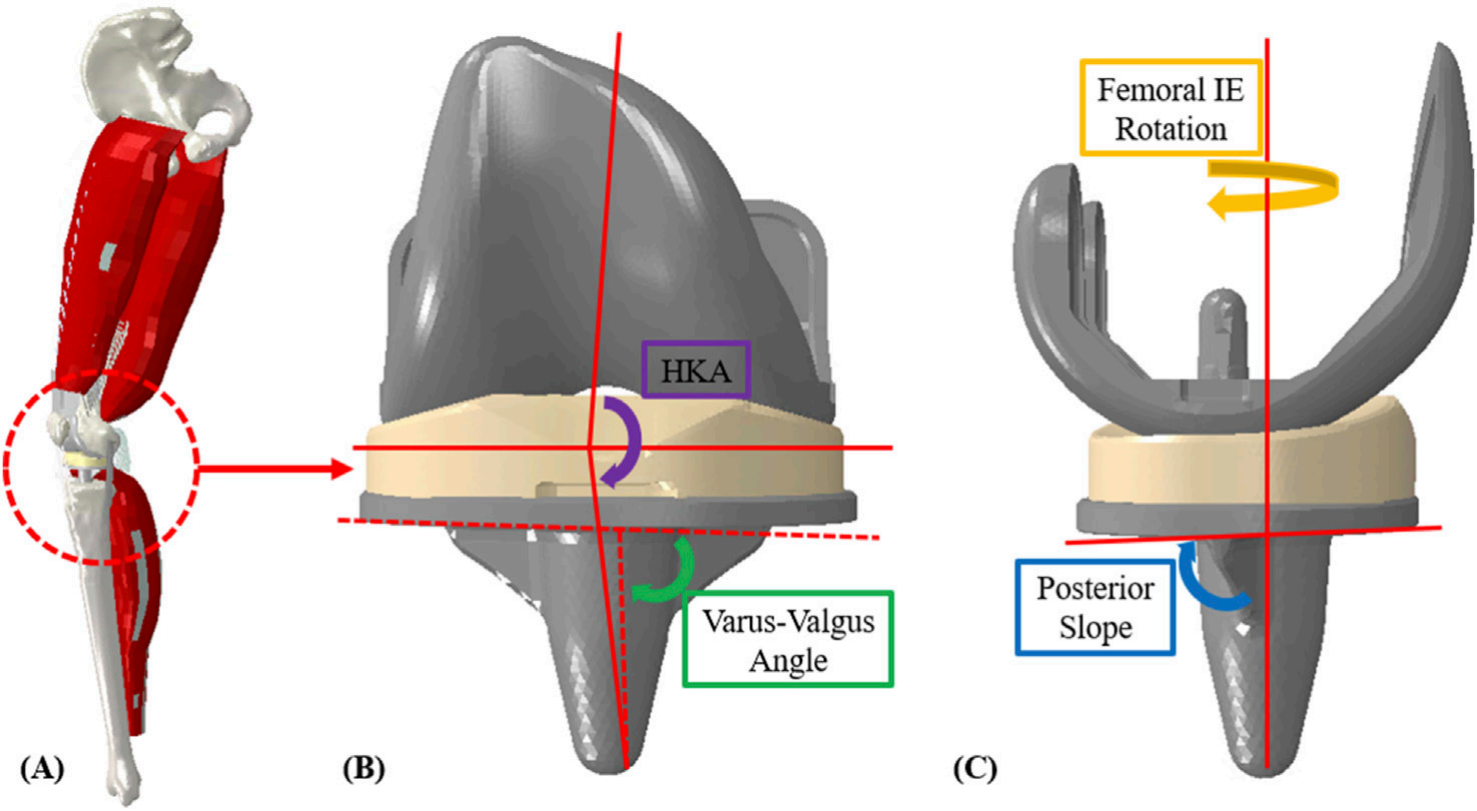
**(A)** Finite element lower limb model; **(B)** coronal implant alignment (HKA and varus-valgus angle); **(C)** sagittal implant alignment (femoral, IE rotation and posterior slope).

Prediction techniques were explored through a dataset of knee joint biomechanics generated via a previously published finite element musculoskeletal lower limb model (Fitzpatrick et al., 2014a). This model applies external boundary conditions at the hip, ankle and quadriceps/hamstrings muscles to estimate dynamic joint level loads, contact mechanics, and kinematics. External loading conditions and surgical alignments were systematically varied to generate a database of linked model inputs and outputs from 1,500 simulations (Figure 1). Machine learning and linear regression models were used to instantaneously estimate the kinematic, kinetic, and contact mechanics output of the lower limb model based on the boundary conditions and surgical alignment.

### Lower limb model

The dynamic finite element analysis used in this study was based on a previously published lower limb model (Fitzpatrick et al., 2014a). Briefly, the model includes the main bones of the lower limb, TKR implants (a contemporary cruciate-retaining (CR) fixed-bearing (FB) TKR), and key muscles and ligaments. The quadriceps and hamstrings are represented with four muscle bundles. Actuators apply external boundary conditions to replicate measured joint level loading from the Orthoload telemetric implant patients for activities of interest (Bergmann et al., 2014). Knee flexion is managed by balancing vertical hip and quadriceps muscle forces, guided by a PID controller. The model was developed in a parameterized manner so that three key sources of variation could be efficiently modified to generate new model instances: external boundary conditions, surgical alignment parameters, and ligament properties.

**FIGURE 2:**
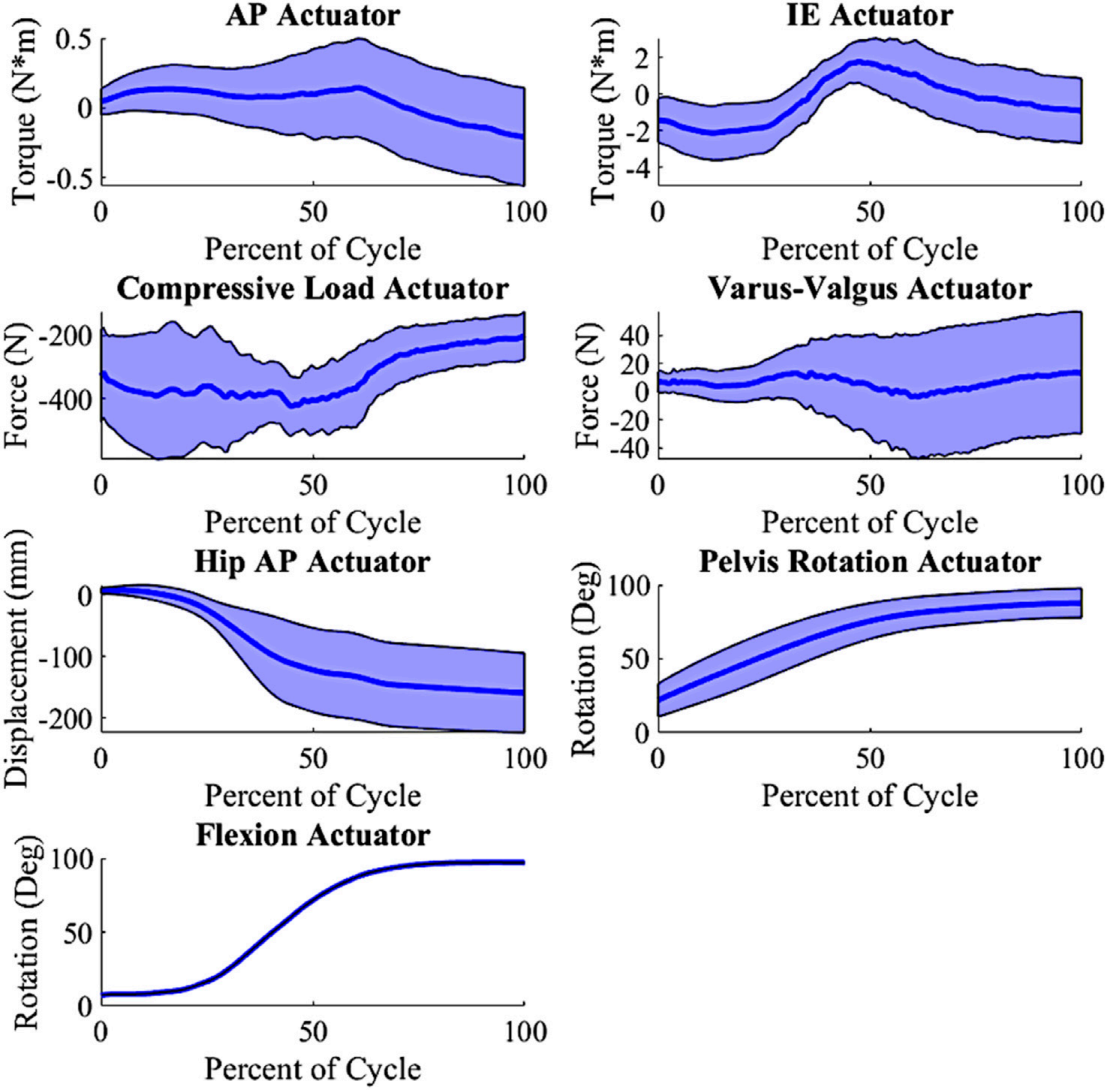
Input actuator distribution, shaded indicate ± 1.0 standard deviation from the mean (wrt to the tibia, +Anterior (Ankle extending); +Internal; +Varus; −Compressive); AP force is created from a torque at the ankle; Varus-Valgus Torque is created via an M-L force at the ankle.

In prior work, the development of the external boundary conditions for nine patients from the Orthoload database was detailed (Maag et al., 2024). Principal component analysis (PCA) was utilized to create new physiologically plausible boundary conditions, maintaining the inherent loading interdependencies of the original external boundary conditions. The PCA inputs consisted of actuator load profiles for each patient during a deep knee bend (DKB) activity cycle. We selected the DKB activity for this study due to its demanding loading and the significant kinematic variability observed in the nine Orthoload patients (Maag et al., 2024). This approach allowed us to expand from the initial set of nine patient-specific models to create new model instances with varied loading conditions. By randomly varying the principal components (PCs) within ±1.5 standard deviations (stdev), we generated 200 unique loading conditions (Figure 2), ensuring substantial variability while maintaining a feasible number for completion.

### Input parameters

Surgical alignment is a key patient-specific factor in determining TKR outcomes. The surgical alignments of interest were varus-valgus (VV) angle, hip-knee-ankle (HKA) angle, posterior slope (PS), and femoral, IE (FIE) rotation. VV angle and HKA were evaluated from −1° (valgus) to 5° (varus), PS from 3° to 7°, and FIE alignment from 0° to 5° (internal) (Figure 1, Table 1). The condition of the collateral ligaments was incorporated through a ±10% variation in slack length for the medial collateral ligament (MCL) and lateral collateral ligament (LCL). Additionally, the insert thicknesses utilized were 5 mm, 8 mm, and 11 mm (with appropriate collateral ligament response) to understand the effects of under/over stuffing the joint space (Table 1). All these parameters are used as inputs to the lower limb model as well as, subsequently, inputs to the prediction methods.

**TABLE 1:** Alignment and condition input parameters.

Input parameter	Range
Varus-Valgus (VV) angle	−1° (Varus) - 5° (Valgus)
Hip-Knee-Ankle angle (HKA)	−1° (Varus) - 5° (Valgus)
Posterior slope (PS)	3°–7°
Femoral internal external (FIE) rotation	0°–5° (internal)
Insert thickness	5 mm, 8 mm, 11 mm
MCL slack length	±10%
LCL slack length	±10%

**FIGURE 3:**
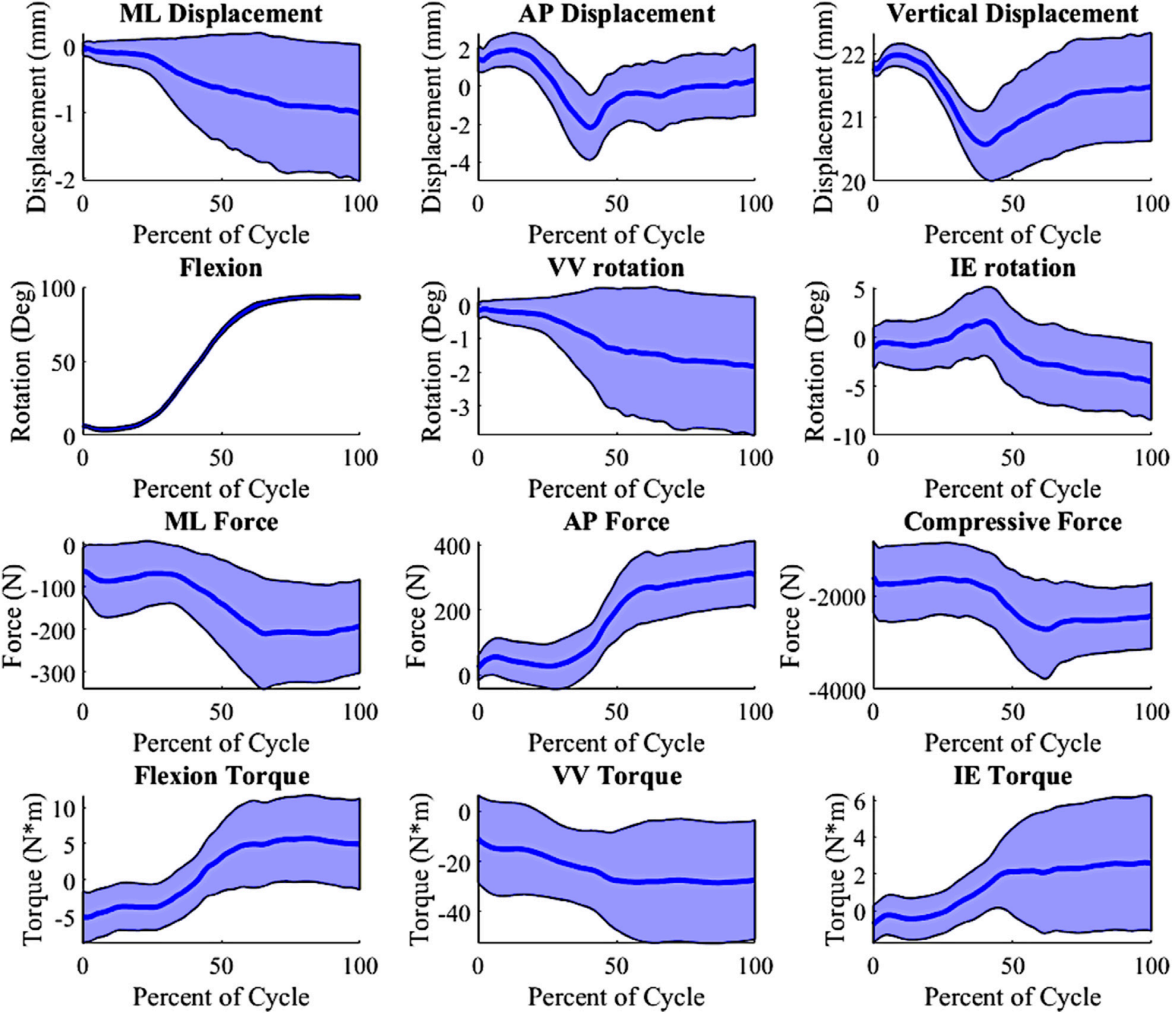
Kinematic and Kinetic output parameter distribution, shaded ±1 distribution (wrt to the tibia, −Medial, +Anterior; +Internal; +Varus; −Compressive).

In-order to create an appropriate number of trials as inputs into the lower limb model, Latin hypercube sampling (LHS) was used. Given the computational expense required for the model, a full parametric approach (e.g., Monte Carlo simulation) would not be practical, so LHS was selected to properly probe the entire design space with a reasonable number of trials. Based on our previous work, we found that Latin Hypercube sampling at 10% of Monte Carlo simulations provides comparable coverage of the design space (Fitzpatrick et al., 2012). Using the defined ranges (Table 1), the LHS algorithm was used to create 500 trials. These 500 trials combined the surgical parameter variation and the 200 loading conditions created from the PCA on the external boundary conditions. The 500 trials were then replicated for the three joint levels/insert thicknesses, creating a total of 1,500 input trials for this DKB activity. The selected surgical parameters and external boundary conditions were combined into inputs for the lower limb model. Combining the alignments and boundary condition created a 14 parameter input file, including joint level, VV angle, HKA angle, PS, FIE rotation, MCL condition and LCL condition. The boundary conditions consisted of the AP actuator, IE actuator, compressive load actuator, varus-valgus actuator, hip AP actuator, pelvis rotation actuator and flexion. A custom-made python script updated the model with each trial’s surgical parameters and boundary conditions and extracted all the kinematics, kinetics and contact mechanics. Simulations ran for approximately 5–6 h using a single core on an Intel XEON Silver 4,116 @ 2.1 GHz with 256 GB of RAM. The outputs combined into 39 total parameters. The 39 parameters consisted of (number of parameters): Grood and Suntay (G&S) kinematics (6) (Grood and Suntay, 1983), G&S kinetics (6), MCL tension, LCL tension, ALS tension, PFL tension, PCL tension, quadriceps load, hamstrings load, medial contact area, lateral contact area, medial center of pressure location (3), medial center of pressure force (3), lateral center of pressure location (3), lateral center of pressure force (3), medial contact pressure peak, medial average contact pressure, medial 90th percentile contact pressure, lateral contact pressure peak, lateral average contact pressure, and lateral 90th percentile contact pressure. Sample output distributions for kinematic and kinetic parameters are shown in Figure 3.

### Data and model preparation

From the 1,500 FE trials, a total of 13 trials were deemed to have un-realistic outcomes (e.g., subluxation) and were discarded, which left 1,487 samples for training/fitting of the prediction methods. Activity cycles were sampled at 181 time increments per trial to allow for adequate resolution. In preparation for training, the inputs and outputs were normalized using the z-score technique (Fei et al., 2021). The prediction techniques selected were the LRM and 4 ML techniques: MLP, biLSTM, CNN, and transformer based. The LRM was selected for its speed, simplicity and to determine if ML based approaches are necessary for this situation. The LRM was created using the fitlm function within MATLAB, using the quadratic option. The data was discretized into 39 by 181 models, to mimic the 39 output variables and 181 time increments of the data. This effectively created 7,059 univariate regression models. The default QR decomposition was used in fitting the least squares problem. The biLSTM was selected for its ability to map both the forward and reverse directions of a time series. LSTM layers are specialized recurrent neural network (RNN) layers that are capable of learning long-term dependencies, especially in long sequences (Sak et al., 2014). A biLSTM is a variant of the LSTM whereas there are two LSTM layers one that learns in the forward direction and the other that learns in the reverse direction. The biLSTM model was constructed from a masking layer four LSTM layers with a width of 181 nodes, and a relu activation layer. This was ordered as masking layer, LSTM (forward), LSTM (reverse), relu activation, LSTM (forward), LSTM (reverse), then terminating in a linear activation layer for regression. Resulting in approximately 937 thousand learnable parameters. Each LSTM forward/reverse pair is what makes the biLSTM its namesake. MLP was chosen as the baseline ML technique as it consisted entirely of full-connected and activation layers. Depth and width was selected to mimic that of the biLSTM. The MLP was constructed using a masking layer, dense layers of 181 width, and a relu activation layer. Similarly to the biLSTM, the layers were ordered as masking layer, two dense layers, relu activation layer, two dense layers, then terminating in a linear activation layer for regression. This resulted in approximately 108 thousand learnable parameters. Both the MLP and biLSTM were implemented through a custom python script using the TensorFlow module. The CNN and Transformer based approaches were implemented through the tsai module for python (Oguiza, 2023). The tsai module is a module built on pytorch that is aimed at making time series machines learning easier to manage. The CNN is an adaptation of the model named InceptionTime that instead of doing classification tasks does regression tasks (Fawaz et al., 2019). This model uses the inception-v4 architecture but for time series, using 1D convolutional layers with residual connections. A depth of 12 layers and a width of 32 was used for this basis of this model. This model has approximately 164 million learnable parameters. The transformer based framework is an adaption of Zerveas et al. to work in a regression based problem (Zerveas et al., 2021). This is a multivariate regression model with positional encoding, 16 attention heads, with a width of 128 hidden nodes and a depth of three layers, resulting in approximately 164 million learnable parameters.

First, the data was shuffled and split randomly into train, validation, and test sets at ratios of 85%, 10% and 5%, respectively. Training, validation and test set distributions were selected based on standard ML practices: it is common place for the data to be split as 70%–90% train, 5%–30% validation, and 5%–15% test (Shahrabadi et al., 2024). This equated to 1,264 samples in the train group, 149 samples in the validation group and 74 samples in the test group. The test group will be used to evaluate the models after the training process is complete. The LRM was fit to only data from the training set to ensure all approaches only had access to the same data. This created a set of linear regression coefficients which were used to predict outcomes in the test dataset. The test set was predicted and the normalized root mean squared error (nRMSE) was calculated. Each ML model was trained using a learning rate of 1 × 10^−5^ using an Adam optimizer and a batch size of 12 (Kingma and Ba, 2014). Batch sizing was optimized for memory usage while learning rate was perturbed until the loss curve was appropriate. Training was terminated once the validation set loss (mean absolute error (MAE)) was no longer reducing (early stopping) or 10,000 epochs was reached. All models were trained on a NVIDIA A6000 chipset. Upon completion of the training, the model switched to inference and used to predict on the test group. The nRMSE was calculated for each of the samples in test group per the model. The average nRMSE for each of the models was used to concluded which of the models more accurately predicted the outputs. Model average nRMSE for each sample in the test group were then compared using the Tukey method with 95% confidence. Additional hyper-parameters were not fully optimized for the models.

## Results

### Training

Time to train the models varied by technique. The MLP took the least amount of time at approximately 3.5 h, the biLSTM took approximately 18 h, while the CNN and transformers took over 24 h (all using a NVIDIA A6000 chipset). Comparatively, the LRM “training” process was much faster than any of the ML techniques at approximately 5 min using one core on a modest CPU. Inference time for all techniques were minimal without any notable time difference.

### Variable prediction

**FIGURE 4:**
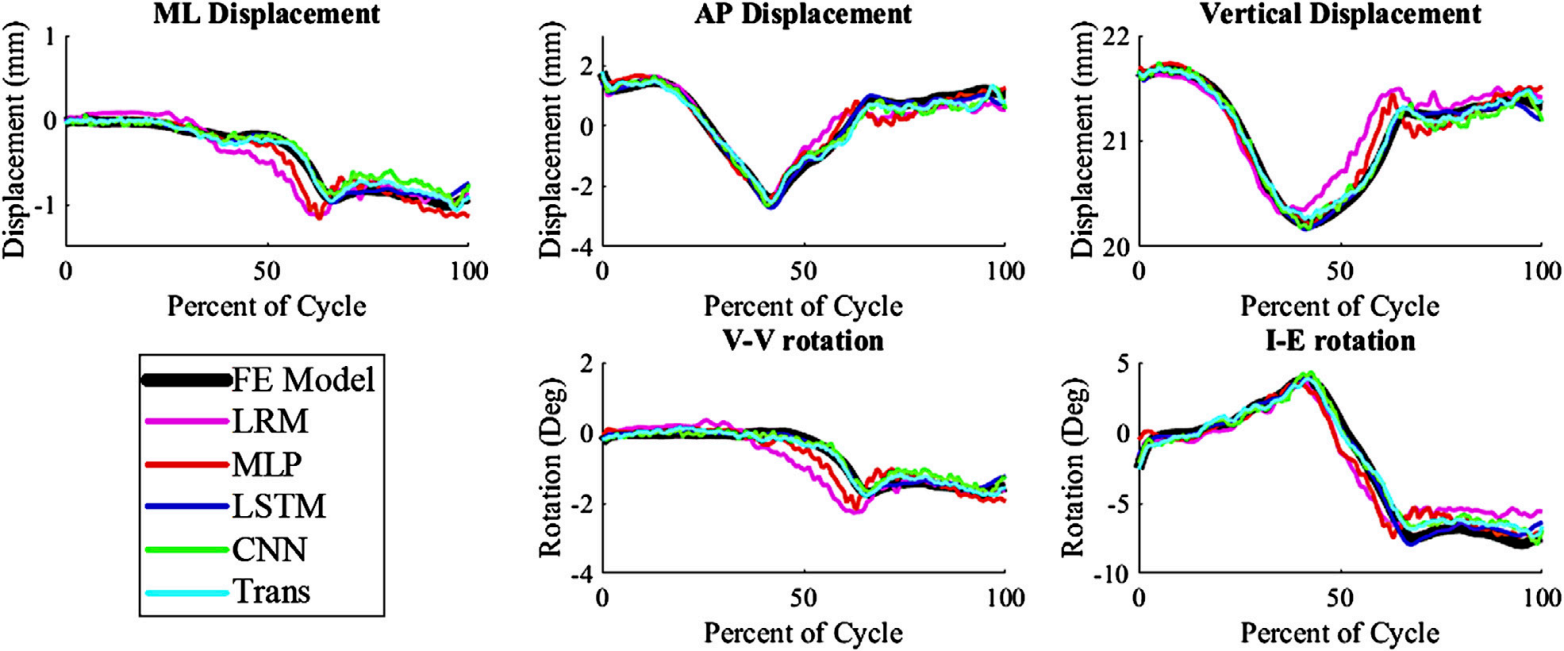
Predicted Kinematics of a test sample with the average nRMSE (biLSTM); (Trans-Transformer); (wrt to the tibia, −Medial, +Anterior; +Internal; +Varus; −Compressive).

**FIGURE 5:**
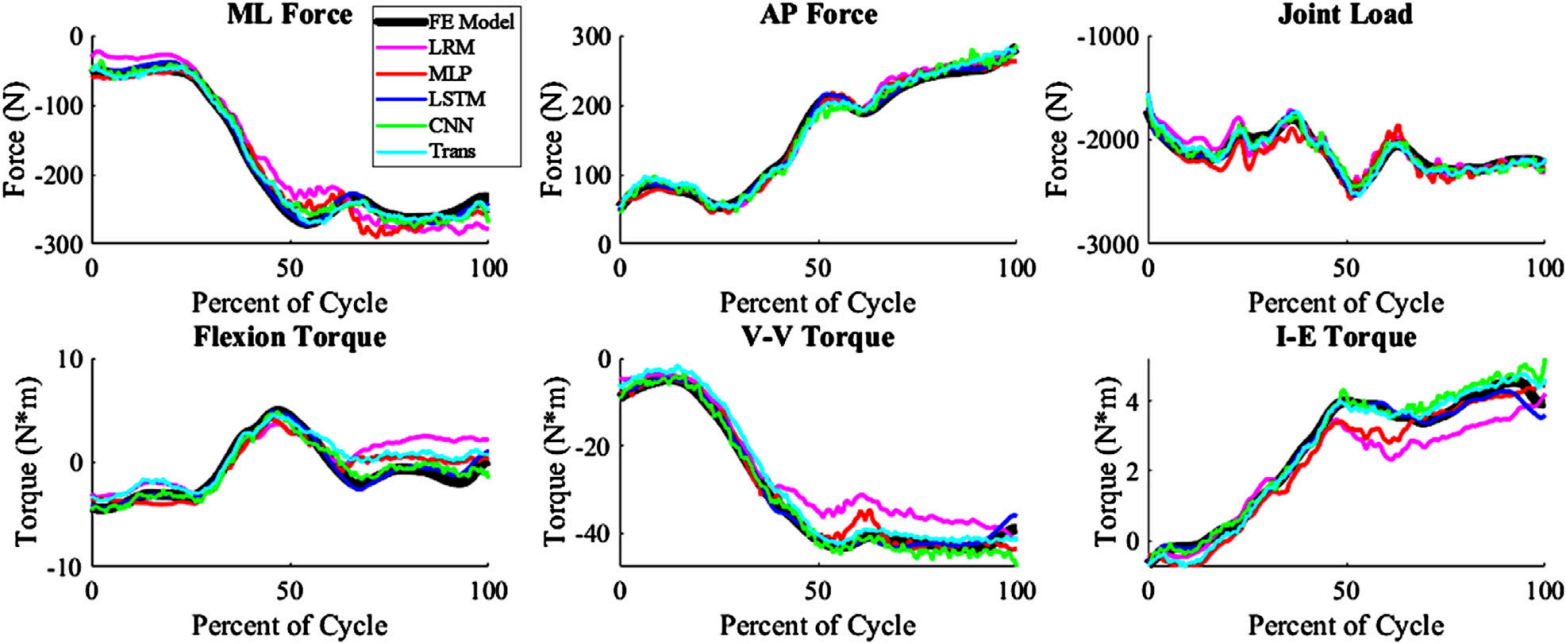
Predicted Kinetics of a test sample with the average nRMSE (biLSTM); (Trans-Transformer); (wrt to the tibia, −Medial, +Anterior; +Internal; +Varus; −Compressive).

**FIGURE 6:**
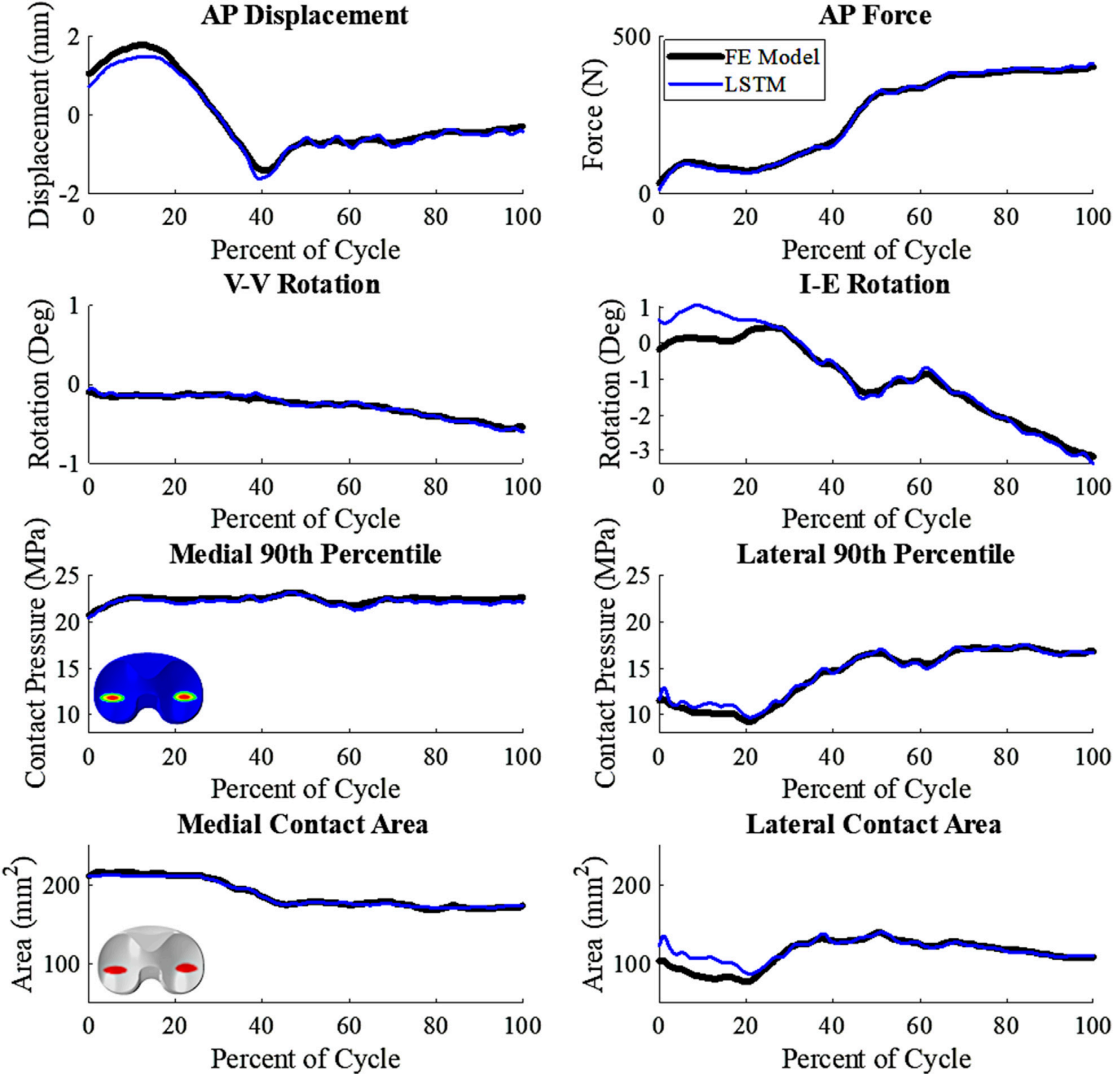
Comparative plot of biLSTM to the FE Model, focusing on key variables of interest. Representative patient of the test cohort, any deviations shown are not systemic (wrt to the tibia, +Anterior; +Internal; +Varus; 90th percentile- 90th percentile contact Pressure).

The kinematics had an average nRMSE of 1.30, 1.15, 0.52, 0.83, and 0.88; and the kinetics had an average nRMSE of 1.00, 0.98, 0.38, 0.63, and 0.71 for the LRM, MLP, biLSTM, CNN, and transformer, respectively (Figures 4, 5). The biLSTM was more than 50% more accurate than the other prediction techniques in all kinematics and kinetics. The biLSTM had limited deviation on an average test sample only accumulating nRMSE of 0.138 and 0.243 in the IE rotation and lateral contact area respectively (Figure 6).

**FIGURE 7:**
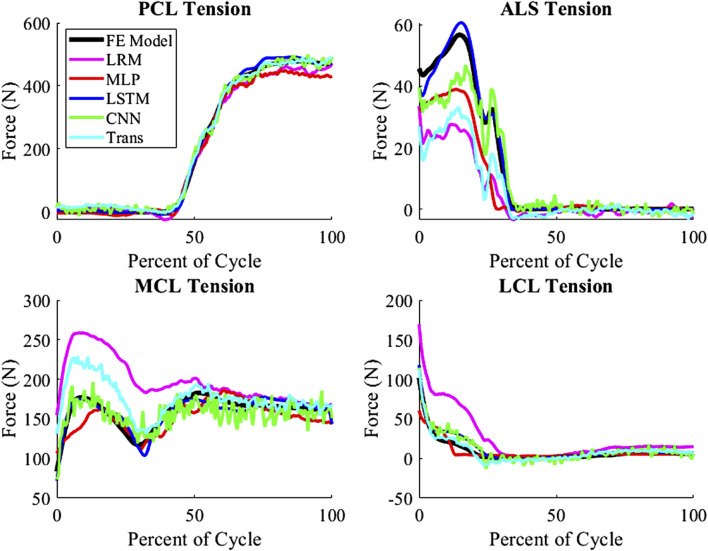
Ligamentous load for average nRMSE sample from the test group (biLSTM); (Trans-Transformer).

The techniques all estimated the tension of PCL, LCL, and MCL accurately, but the ALS estimates were the least accurate among the ligament tensions (Figure 7). The ALS had the highest nRMSE of all the predicted variables, with an average nRMSE of 30.72. Comparatively, the average nRMSE of the PCL, MCL, and LCL was 5.30, 5.73, and 12.49, respectively. In comparison of the prediction technique, the biLSTM outperformed all of techniques in predicting ligamentous loads by at least 50%.

### Model performance

**FIGURE 8:**
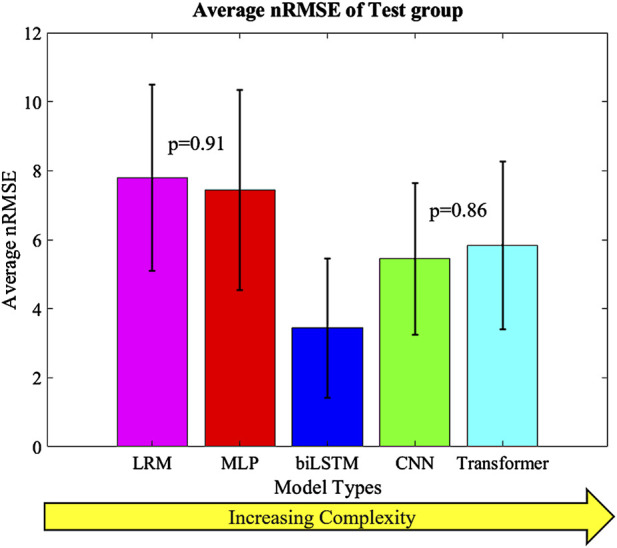
Average nRMSE of the test groups with increasing complexity. Error bars = ±1SD; CNN and Transformer (p = 0.86) as well as MLP and LRM (p = 0.91) were not statistically different.

**FIGURE 9:**
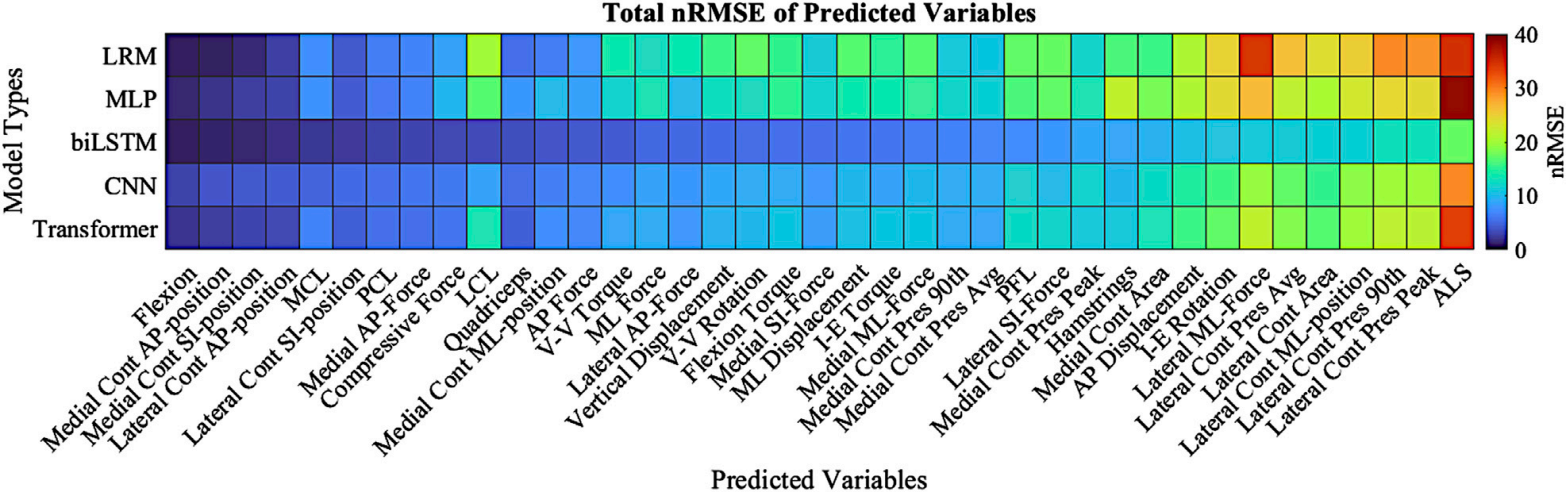
Total nRMSE of predicted variables; variables are ranked in highest nRMSE (right) down to the lowest nRMSE (left). Dark Red denotes higher nRMSE with dark blue being the lowest. Cont-(Contact), Pres-(Pressure), 90th-(90th Percentile).

The highest overall accuracy of the models was the biLSTM with an average nRMSE of 3.4 (Figure 8). This was a reduction in nRMSE of 55.8%, 53.8%, 36.8%, and 41.1% over the LRM, MLP, CNN, and Transformer, respectively. Differences in the nRMSE were analyzed for statistical significance using a tukey pairwise comparison with 95% confidence intervals—the LRM and MLP as well as the CNN and transformer comparison showed no statistical difference. The performance of each model to predict the individual outputs of the model was calculated by summing the nRMSE of each test sample per variable (Figure 9).

## Discussion

This study explored analytical and ML models to instantaneously estimate knee joint biomechanics from a FE lower limb model. The models input joint alignment and ligament data, and external boundary conditions to output kinematics, kinetics and contact mechanics. The models tested in this study were LRM, MLP, biLSTM, CNN and transformer based. The biLSTM was the most accurate model as measured by average nRMSE, and it was significantly lower than the other three models according to the Tukey method with 95% confidence intervals. These models can obviously infer much faster than an FE model or a rigid body dynamics solution, allowing for almost immediate insight into the TKR system. Moreover, the models were all relatively small, which reduced memory and computation requirements. This small size makes them suitable for edge devices similar to those used in the operating room. These kinds of approaches could eventually enable real-time feedback for patient alignment and ligament balancing.

The prediction techniques used in the study had different levels of complexity. It was expected that more complexity would result in better prediction of the output variables, but this was not the case. Complexity is not always a measure of the learnable parameters in a model, in the case of the CNN and transformer based models the complexity differences are based on their construction and branding architecture. The biLSTM, which has a medium level of complexity, performed the best in predicting the output variables. It is possible this could have been improved by optimizing the hyper-parameters of each technique for this specific scenario, but this was not the focus of this proof-of-concept study. The models already exhibit good performance, so hyper-parameter optimization was not pursued due to the effort required and the minimal potential improvements in performance. The CNN and transformer, which were more complex, may not have been necessary for this system as, the CNN and transformer tend to focus on detail rather than generalizing as the biLSTM does. This can be seen in their output, which has more noise and follows the sharper changes in some of the data (Figures 4, 5).

The activity predicted in this study was the DKB, and it was chosen as it has a large variability in the output kinetics, kinematics and contact mechanics (Figure 3). It was assumed that this level of variability would provide a level of complexity of that would properly test the predictive models. While all the models were able to predict relatively well within this activity, it is unknown how well this would translate to other activities. However, given the level accuracy of the models for the DKB activity it is expected that these models would do well at predicting other activities as well. Future studies will investigate the predictive power of these models with other activities.

This study shows promise for estimating joint-level TKR mechanics instantaneously for future application in the operating room. Given the quality of real-time predictions, as all the techniques represent the dynamic mechanical system fairly well, the finite element model and any associated inaccuracy remains as the primary limitation in moving forward. The focus of this study was to quantify how well this ML approach could represent the results from an FE model. Although sufficient breath and volume of *in vivo* data was not available, as such data does become more commonplace, we can apply the same methodology to capture the *in vivo* behavior. In this study, adapting the lower limb to best fit a patient through geometry, properties, and loading conditions were not addressed. Development and validation of this complete process for estimation of joint mechanics will be required and remains a significant hurdle. Once complete, a more comprehensive pre-run simulation database may be developed.

The utilization of a finite element model of the lower limb as a data generation tool for training various predictive models, including the notably effective biLSTM model, underscores the potential of these technologies in optimizing surgical outcomes. The ability to provide real-time feedback during total knee replacement surgeries could enhance surgical decision-making, leading to improved patient outcomes. We think this real-time data could enhance alignment optimization to achieve ideal kinematics and kinetics, minimize micromotion in cementless TKR, and reduce fixation stresses in cemented TKR. For instance, optimizing implant I-E rotation and A-P position could help maintain natural knee joint movements, thereby reducing soft tissue strain in the implanted joint. This study’s findings advocate for the integration of advanced machine learning techniques into clinical practices, while emphasizing the need for further research to overcome current limitations and enhance model generalizability.

## Data Availability

The datasets presented in this article are not readily available because data subject to commercial research agreement. Requests to access the datasets should be directed to paul.rullkoetter@du.edu.
